# Understanding the Mesothelioma Journey from Diagnosis through Bereavement

**DOI:** 10.1093/geroni/igaf122.4039

**Published:** 2025-12-31

**Authors:** Amanda Woodward, Jenavry Hirsch, Xuehan Zhang, Maria Towe, Claudine Benmar, Julie White, Brian Lapacek, Melissa Culligan

**Affiliations:** Michigan State University, East Lansing, Michigan, United States; Michigan State University, East Lansing, Michigan, United States; Michigan State University, East Lansing, Michigan, United States; Mesothelioma Applied Research Foundation, Washington, District of Columbia, United States; Mesothelioma Applied Research Foundation, Washington, District of Columbia, United States; Mesothelioma Applied Research Foundation, Washington, District of Columbia, United States; Mesothelioma Applied Research Foundation, Washington, District of Columbia, United States; Temple University Hospital, Philadelphia, Pennsylvania, United States

## Abstract

The Michigan State University School of Social Work (MSUSSW) and the Mesothelioma Applied Research Foundation (Meso Foundation) are collaborating on a federally funded four-year project to develop and test an online support intervention for those caring for a loved one with mesothelioma. This poster will present results from the first phase of this work within the context of a community-engaged research framework. Twenty-seven semi-structured interviews (5 patients, 12 caregivers, 10 bereaved) were conducted via Zoom between December 2024 and May 2025. Interview topics included the diagnosis, the treatment journey, the experience of providing care, decision making, planning for end of life, and caregiving and bereavement support. Interviews were recorded and transcribed. A codebook was created through an iterative process and each interview was coded in Dedoose by two team members with discrepancies and questions resolved by the full team. Initial thematic analysis identified three main findings. First the uniqueness of mesothelioma compared to other cancer experiences permeated all aspects of caregiving. Second, caregivers and patients experienced a lack of control. For example, limited treatment options meant there were really no treatment decisions to make. Third the impact of health-related social factors was heightened because of the unique aspects of mesothelioma (for example, the need to travel to receive care from experts). Based on these findings, the next phase of this research will include mapping the trajectory from diagnosis through bereavement to identify key time points for intervention and confirming these findings through focus group discussions.

